# Efficacy of a vegetal mixture composed of *Zingiber officinale*, *Echinacea purpurea*, and *Centella asiatica* in a mouse model of neuroinflammation: *In vivo* and *ex vivo* analysis

**DOI:** 10.3389/fnut.2022.887378

**Published:** 2022-08-30

**Authors:** Laura Micheli, Alessandra Toti, Elena Lucarini, Valentina Ferrara, Clara Ciampi, Guendalina Olivero, Anna Pittaluga, Luisa Mattoli, Caroline Pelucchini, Michela Burico, Jacopo Lucci, Donatello Carrino, Alessandra Pacini, Stefano Pallanti, Lorenzo Di Cesare Mannelli, Carla Ghelardini

**Affiliations:** ^1^Neurofarba—Pharmacology and Toxicology Section, Department of Neuroscience, Psychology, Drug Research and Child Health, University of Florence, Florence, Italy; ^2^Department of Pharmacy, School of Medical and Pharmaceutical Sciences, University of Genoa, Genoa, Italy; ^3^Centre of Excellence for Biomedical Research, University of Genoa, Genoa, Italy; ^4^Innovation and Medical Science Division, Aboca SpA Società Agricola, Sansepolcro, Italy; ^5^Anatomy and Histology Section, Department of Experimental and Clinical Medicine, University of Florence, Florence, Italy; ^6^Psychiatry Section, Department of Neurofarba, University of Florence, Florence, Italy; ^7^Department of Psychiatry and Behavioral Sciences, Albert Einstein College of Medicine, New York, NY, United States; ^8^Institute of Neuroscience, Florence, Italy

**Keywords:** LPS, neuroinflammation, vegetal mixture, glial cells, pain, mood disorders, CNS complement proteins

## Abstract

Experimental evidence suggests that neuroinflammation is a key pathological event of many diseases affecting the nervous system. It has been well recognized that these devastating illnesses (e.g., Alzheimer’s, Parkinson’s, depression, and chronic pain) are multifactorial, involving many pathogenic mechanisms, reason why pharmacological treatments are unsatisfactory. The purpose of this study was to evaluate the efficacy of a vegetal mixture capable of offering a multiple approach required to manage the multifactoriality of neuroinflammation. A mixture composed of *Zingiber officinale* (150 mg kg^−1^), *Echinacea purpurea* (20 mg kg^−1^), and *Centella asiatica* (200 mg kg^−1^) was tested in a mouse model of systemic neuroinflammation induced by lipopolysaccharide (LPS, 1 mg kg^−1^). Repeated treatment with the vegetal mixture was able to completely counteract thermal and mechanical allodynia as reported by the Cold plate and von Frey tests, respectively, and to reduce the motor impairments as demonstrated by the Rota rod test. Moreover, the mixture was capable of neutralizing the memory loss in the Passive avoidance test and reducing depressive-like behavior in the Porsolt test, while no efficacy was shown in decreasing anhedonia as demonstrated by the Sucrose preference test. Finally, LPS stimulation caused a significant increase in the activation of glial cells, of the central complement proteins and of inflammatory cytokines in selected regions of the central nervous system (CNS), which were rebalanced in animals treated with the vegetal mixture. In conclusion, the vegetal mixture tested thwarted the plethora of symptoms evoked by LPS, thus being a potential candidate for future investigations in the context of neuroinflammation.

## Introduction

Neuroinflammation is a pivotal factor in the pathogenesis of many neurodegenerative diseases (1) and psychiatric illnesses like depression (2), Parkinson’s disease (PD) (3), Alzheimer’s disease (AD), (4), Huntington’s disease (5), and multiple sclerosis (6). Moreover, it has been implicated in sickness behavior (7), reduced cognition (8), and memory (9), as well as in age-related increased sensitization of the immune system to intrinsic and extrinsic stimuli (10, 11). Lastly, it has been demonstrated that the coronavirus 2019 (COVID-19) infection has triggered and reinforced obsessive and compulsive behavior leading to an aggravation of anxiety and depressive symptoms in some patients (12, 13). This could be partially explained due to an increase in the levels of proinflammatory chemokines and cytokines that are responsible for the pathophysiology of comorbid neuropsychiatric manifestations (14, 15). Also, neuropathic pain, caused by different etiology, is characterized by excessive inflammation in both the peripheral and central nervous systems (CNS), which may contribute to the initiation and maintenance of persistent pain (16). Overall, neuroinflammation can be considered as a normal response that origins in order to maintain homeostasis in the nervous system. This response involves the synthesis and release of molecules endowed with inflammatory properties; however, a neuroinflammatory process that is too strong or protracted becomes maladaptive, leading to cellular dysfunction which is seen during stress, chronic pain, and chronic neurodegeneration (17).

In this scenario, effective therapies are lacking, and a relatively dry CNS drug pipeline poses a formidable challenge to pharmacologists who have to develop drugs that can counteract the multitude of brain diseases and symptoms, such as cognitive impairment (from mild to more severe), motor impairment, memory loss, mood alterations, and pain. Indeed, the use of anti-inflammatory drugs like non-steroidal anti-inflammatory drugs (NSAIDs) can ameliorate some symptoms and reduce the extent of neurodegeneration (18); however, the primary mechanism of neuroinflammation is not well-understood and new therapeutic approaches are mandatory to provide significant relief for these patients.

Traditional medicine has been based on the use of medicinal plants for millennia, and mankind has been educated from experience and observation to use plants correctly, strengthening an extensive culture of their use. Indeed, phytotherapeutic preparations can offer a multiple approach to the multifactorial nature of neuroinflammation relying on the complex mixture of one or more plants (19).

*Zingiber officinale*, *Centella asiatica*, and *Echinacea purpurea* are plants rich in bioactive compounds with several pharmacological effects. *Zingiber officinale* Roscoe (ginger), belonging to the Zingiberaceae family, has been commonly consumed as a spice and used as herbal medicine for a long time (20). From a pharmacological point of view, ginger demonstrated to share with NSAIDs the properties of inhibiting prostaglandin synthesis and to be dual inhibitors of cyclooxygenase-2 (COX-2) and lipoxygenase (LOX) enzymes; therefore, it can effectively reduce the synthesis of both prostaglandins (PGs) and leukotrienes (LTs) (21). Additionally, it can also reduce the synthesis of proinflammatory cytokines, such as TNF-α, IL-1, and IL-8 (22, 23). These preclinical findings were supported by a study in which ginger reduced TNF-α and hs-C-reactive protein (CRP) levels in patients with diabetes (24). Moreover, ginger showed protective properties against rheumatic diseases, *via* rebalancing musculoskeletal disorders and joint inflammation-inducing T-helper-2 and anti-inflammatory cytokine production (e.g., IL-4 and IL-10) (25); decreasing IL-2, IL-6, and IL-1β cytokine levels; and inhibiting the release of substance P as pain and inflammation mediator (26). The beneficial effects of ginger have been mainly attributed to the presence of gingerols and shogaols (phenolic compounds) (27).

*Echinacea purpurea* (L.) Moench is an endemic plant in the US Great Plains and in the Canadian prairies of North America. Even though Echinacea originates in North America, the plant’s beneficial effects were discovered by Europeans who used it for its immunomodulatory properties. The bioactive constituents are mainly phenols, which are reported to exert neuroprotective effects by reducing apoptotic markers (28), polar caffeic acid derivatives (cicoric acid and echinacoside), and lipophilic alkamides (29, 30). Alkamides, in early investigations, have been demonstrated to evoke stimulatory effects on phagocytosis, and these constituents have been found in relevant concentrations in the human blood after the oral intake of Echinacea extract. Alkamides share similarities with anandamide, the endogenous ligand of cannabinoid (CB) receptors, and consequently bind to CB receptors, which are supposed to be the theoretical mechanism of action of Echinacea alkamides as immunomodulatory agents (31). In addition, *Centella asiatica* was successfully used for a long time in traditional Chinese medicine as a nerve tonic (32). The bioactive micronutrients are mainly asiatic acid, asiaticoside, madecassoside, and madecassic acid and are reported to be effective in retarding brain aging and assisting in the renewal of the neural tissue; therefore, it is effective in improving memory, increasing attention, and concentration (33). *Centella asiatica* has also shown an anti-hyperalgesic and protective profile in a rat model of osteoarthritis when the extract of this plant was intra-articularly injected (34). Despite the beneficial effects of these plants, as previously reported, no preclinical data are available about the use of a mixture of these three plants against the plethora of neuroinflammation symptoms.

The aim of this study was to highlight the efficacy of a repeated treatment with a vegetal mixture composed of *Zingiber officinale*, *Echinacea purpurea*, and *Centella asiatica* in a mouse model of neuroinflammation obtained by the sub-chronic injection of LPS. In particular, we focused our attention on a plethora of neuroinflammatory symptoms, such as memory impairment, depression, anhedonia, pain, and motor alterations. Moreover, *ex vivo* analyses were performed to determine the effect of the treatment on astrocytosis and microgliosis in the spinal cord and prefrontal cortex (PFC) and on the selected proteins of the complement system in the PFC.

## Materials and methods

### Animals

Eight- to ten-week-old male CD-1 mice (Envigo, Varese, Italy) weighing approximately 20–25 g were used for all the experiments described in the present study. Animals were housed in CeSAL (Centro Stabulazione Animali da Laboratorio, University of Florence) and used at least 1 week after their arrival. Eight mice were housed per cage (size 26 × 41 cm) kept at 23 ± 1°C with a 12-h light/dark cycle, the light at 7 a.m., and were fed a standard laboratory diet and tap water *ad libitum*.

All animal manipulations were carried out according to the Directive 2010/63/EU of the European Parliament and the European Union Council (22 September 2010) on the protection of animals used for scientific purposes. The ethical policy of the University of Florence complies with the Guide for the Care and Use of Laboratory Animals of the US National Institutes of Health (NIH Publication No. 85–23, revised 1996; University of Florence assurance number: A5278-01). Formal approval to conduct the experiments described was obtained from the Animal Subjects Review Board of the University of Florence. Experiments involving animals have been conducted according to the ARRIVE guidelines (35). All efforts were made to minimize animal suffering and reduce the number of animals used.

### Lipopolysaccharide-induced neuroinflammation

Lipopolysaccharide (LPS, 1 mg kg^–1^; Sigma-Aldrich, St. Louis, MO, United States) was dissolved in sterile saline solution and intraperitoneally injected on four alternate days (days 1, 3, 5, and 8). Behavioral measurements were started from day 9. Control animals were treated with a vehicle.

### Plant materials

Vegetal extracts were supplied by ABOCA (Sansepolcro, Italy). *Zingiber officinale* Roscoe Rhizoma was subjected to extraction by using 30% ethanol (ethanol: water, 30:70 v/v) for 8 h at 50°C and filtered to remove solid exhausted material. The resulting clarified extract was concentrated by ethanol evaporation under vacuum, until reaching the concentration factor of 10:1 (v:v, initial alcoholic extract volume compared to the volume after the evaporation step), and then freeze-dried for 72 h. The resulting extract was stored until use at 4°C, away from light and humidity. The flowering tops of *Echinacea Purpurea* were subjected to extraction by using 45% ethanol (ethanol: water, 45:55 v/v) for 8 h at 50°C and filtered to remove solid exhausted material. The resulting clarified extract was concentrated by ethanol evaporation under vacuum, until reaching the concentration factor of 8:1 (v:v, initial volume of alcoholic extract compared to the volume after evaporation step), and then freeze-dried for 72 h. The resulting extract was stored until use at 20°C, away from light and humidity. *Centella asiatica* extract was prepared following the protocol described by Micheli et al. (34). The characterization of all extracts was also performed by ABOCA. Tables 1, 2 present the characterization of Echinacea and Zingiber, respectively. *Centella asiatica* was characterized and described previously (34). Zingiber characterization was performed according to the methods reported in USP-NF.

**TABLE 1:** Characterization of *Echinacea purpurea* extract.

Classes of compounds		% (g of compound/100 g of sample)		
**Phenols, total**				**5.945**
Of which phenylpropanoids			0.037	
	Echinacoside	0.000		
	Chlorogenic acid	0.037		
Of which phenolic acids			5.908	
	Caftaric acid	2.083		
	Cicoric acid	3.825		

**TABLE 2:** Characterization of *Zingiber officinale* extract.

Classes of compounds		% (g of compound/100 g of sample)	
**Gingerols, totals**			**3.107**
	6-Gingerol	2.539	
	8-Gingerol	0.339	
	10-Gingerol	0.229	
**Shogaols, totals**			**0.647**
	6-Shogaol	0.647	

Echinacea purpurea was characterized by means of a reverse-phase HPLC-UV method, which involved the sample extraction with 37% ethanol: water: hydrochloric acid (44.5:54.5:1.0, v/v and) in an ultrasonic bath at 35°C. The corresponding solution was filtered and analyzed by means of an HPLC-DAD at 330 nm. The mobile phase was ultrapure water acidified with 0.2% phosphoric acid (solvent A) and acetonitrile (solvent). The program involved a linear gradient elution with solvent A from 90% to 80% and solvent B from 10% to 20% for 40 min. At 40.5 min solvent A was 10% and solvent B from 90% and remained in these conditions for 5 min. Caftaric acid, chlorogenic acid, echinacoside, and chicoric acid were eluted at 9.6, 10.7, 22.4, and 39.5 min, respectively. Quantitative data were obtained using the external regression curve method.

### Treatment

The plant extracts of *Zingiber officinale* (150 mg kg^–1^), *Echinacea purpurea* (20 mg kg^–1^), and *Centella asiatica* (200 mg kg^–1^) were suspended in 1% carboxymethylcellulose sodium salt (CMC; Sigma-Aldrich, St. Louis, MO, United States) and orally administered daily starting from day 1 till the end of the experiment (day 12). Control animals received an equal volume of vehicle. Behavioral measurements were conducted from days 9 to 12 when animals were sacrificed for the *ex vivo* analyses.

### von Frey test

The animals were placed in 20 × 20 cm Plexiglas boxes equipped with a metallic mesh floor, 20 cm above the bench. A habituation of 15 min was allowed before the test. An electronic von Frey hair unit (Ugo Basile, Varese, Italy) was used, and the withdrawal threshold was evaluated by applying force ranging from 0 to 5 g with a 0.2 g accuracy. Punctuate stimulus was applied to the mid-plantar area of each anterior paw from below the meshy floor through a plastic tip, and the withdrawal threshold was automatically displayed on the screen.

The paw sensitivity threshold was defined as the minimum pressure required to elicit a robust and immediate withdrawal reflex of the paw. Voluntary movements associated with locomotion were not taken as a withdrawal response. Stimuli were applied on each anterior paw with an interval of 5 s. The measurement was repeated five times, and the final value was obtained by averaging the five measures (36, 37).

### Cold plate test

Thermal allodynia was assessed using the Cold plate test. With minimal animal–handler interaction, mice were taken from home cages and placed onto the surface of the cold plate (Ugo Basile, Varese, Italy) maintained at a constant temperature of 4°C ± 1°C. Ambulation was restricted by a cylindrical Plexiglas chamber (diameter, 10 cm; height, 15 cm) with an open top. A timer controlled by a foot peddle was used to monitor timing response latency from the moment the mouse was placed onto the cold plate. Pain-related behavior (licking of the hind paw) was observed, and the time (seconds) of the first sign was recorded. The cutoff time of the latency of paw lifting or licking was set at 30 s (38, 39).

### Passive avoidance test

We followed the protocol of the step-through method described previously (40). The apparatus consisted of a two-compartment acrylic box with a lighted compartment connected to a darkened one by a guillotine door. As soon as the mouse entered the dark compartment, it received a punishing electrical shock (0.5 mA, 1 s). The latency times for entering the dark compartment were measured during the training test and after 24 h of the retention test. The data recorded during the retention session are reported in the “Results” section. The maximum entry latency allowed in the training and retention sessions was 60 and 180 s, respectively.

### Porsolt test

The forced swimming test used was similar to the method described previously (41). Briefly, mice were placed individually in glass cylinders (height: 25 cm, diameter: 10 cm) containing 12 cm of water maintained at 22–23°C and left there for 6 min. A mouse was considered to be immobile when it floated in the water, in an upright position, and made only small movements to keep its head above water. The duration of mobility was recorded during the last 4 min of the 6-min test. An increase in the duration of immobility is indicative of a depressant-like effect (42).

### Sucrose preference test

Sucrose preference was examined as a test to assess anhedonia. Mice were placed in cages equipped with two bottles: a bottle containing 2% sucrose solution and a bottle containing water for 24 h after the beginning of the experiment. The preference index was calculated according to the following formula: preference index = volume of consumed sucrose solution/(volume of consumed sucrose solution + volume of consumed water).

### Rota rod test

The apparatus consisted of a base platform and a rotating rod with a diameter of 3 cm and a non-slippery surface. The rod was placed at a height of 15 cm from the base. The rod, 30 cm in length, was divided into five equal sections by six disks. Thus, up to five mice were tested simultaneously on the apparatus, with the rotating speed of the rod at 16 revolutions per minute. The integrity of motor coordination was assessed on the basis of the number of falls from the rod in 10 min (43).

### Tissue collection

At the end of the behavioral experiments, animals were killed by decapitation. The lumbar spinal cord was collected, and one part was fixed by immersion in 4% neutral buffered formalin and one part was frozen using liquid nitrogen. The prefrontal cortex (PFC) was also collected and frozen using liquid nitrogen.

### Enzyme-linked immunosorbent assay

The prefrontal cortex and spinal cord samples were homogenized (100 mg of tissue/mL of cold PBS). The samples were centrifuged at 12,000 × *g* for 15 min at 4°C. The supernatant was collected for protein quantification using a bicinchoninic acid (BCA) protein assay reagent kit (Merck, Milan, Italy). The levels of IL-1β (Thermo Fisher Scientific, Milan, Italy) and TNF-α (Thermo Fisher Scientific, Milan, Italy) in the PFC and spinal cord were measured using enzyme-linked immunosorbent assay (ELISA) kits according to the manufacturer’s instructions. The content of cytokines is expressed as pg of cytokines/mg of protein.

### Western blot analysis of prefrontal cortex lysates

Mouse PFC samples were homogenized in modified RIPA buffer (10 mM Tris, pH 7.4, 150 mM NaCl, 1 mM EDTA, 0.1% SDS, 1% Triton X-100, 1 mM sodium orthovanadate, and protease inhibitors), sonicated, and centrifuged at 20,000 × g for 10 min at 4°C. The supernatant was kept for the immunoblot analysis and quantified for protein content with a BCA assay. Samples were boiled for 5 min at 95°C, separated by SDS-10% PAGE (30 μg/lane), and then blotted onto the PVDF membrane. Membranes were blocked for 1 h at room temperature with Tris-buffered saline Tween (t-TBS: 20 mM Tris, pH 7.4, 150 mM NaCl, and 0.05% Tween 20) containing 5% (w/v) non-fat dried milk, and then probed with the following primary antibodies overnight at 4°C: rabbit anti-CD11b (1:1,000, ab200999, Abcam, Cambridge, United Kingdom), mouse anti-GFAP (1:10,000, G3893, Sigma, St. Louis, MO, United States), mouse anti-C1q (1:25, Abcam, ab71940), rabbit anti-C3 (1:1,000, Abcam, ab200999), and mouse anti-β-actin (1:5,000, Sigma). After extensive washes in t-TBS, membranes were incubated for 1 h at room temperature with the appropriate horseradish peroxidase-linked secondary antibodies (1:20,000, A9044 and A9169, Sigma). Immunoblots were visualized with an enhanced chemiluminescence (ECL) Western blotting detection system. Images were acquired using the Alliance LD6 images capture system (Uvitec, Cambridge, United Kingdom) and analyzed using UVI-1D software (Uvitec, Cambridge, United Kingdom).

### Immunohistochemistry of the spinal cord

On day 12, after behavioral measurements, mice were sacrificed, and the lumbar spinal cord segments were removed, post-fixed in 4% paraformaldehyde, and then cryoprotected in 30% sucrose solution at 4°C. Slide-mounted cryostat sections (5 μm) were processed for indirect immunofluorescence histochemistry.

Formalin-fixed cryostat sections (5 μm) were incubated for 30 min in a ready-to-go blocking solution (Bio-Optica, Milan, Italy) at room temperature to block unspecific binding. The primary antibodies, incubated overnight at 4°C, were directed against Iba1 (rabbit, 1:200; Wako Chemicals, Richmond, United States) for microglial staining or against the glial fibrillary acidic protein (GFAP; rabbit, 1:200; Dako, United States) for astrocyte staining. After rinsing in PBST, sections were incubated in donkey anti-rabbit IgG secondary antibody labeled with Alexa Fluor 488 (1:500, Invitrogen, Milan, Italy) at room temperature for 1 h. Nuclei were stained with 4’,6-diamidin-2-fenilindolo (DAPI, 1:2,000; Invitrogen, Milan, Italy).

Negative control sections (no exposure to the primary antisera) were processed concurrently with the other sections for all the immunohistochemical studies. We obtained a single optical density value for the dorsal horns by averaging the two sides in each mouse, and these values were compared to the homologous average values from the vehicle-treated animals.

Images were acquired by using a motorized Leica DM6000B microscope equipped with a DFC350FX camera (Leica, Mannheim, Germany). Microglia and astrocyte morphology was assessed by inspecting at least three fields (×40, 0.75NA objective) in the dorsal horn.

Quantitative analysis of GFAP and Iba1-positive cells was performed by viewing at least three independent fields through a ×40 0.5NA objective. Immunofluorescent staining was measured as mean fluorescence intensity using ImageJ software (ImageJ, National Institute of Health, United States)^1^ by an automatic thresholding algorithm. Furthermore, GFAP or Iba1-positive cells were quantified by thresholding automatic count. The results, given as mean fluorescent intensity (arbitrary units) by the thresholded fluorescent signal, revealed a common trend between GFAP/Iba1 expression and astrocyte/microglial cell number (data not shown). Five spinal cord sections were analyzed for each animal.

### Statistical analysis

Researchers not informed about the specific treatment of each animal group carried out the tests. Results were expressed as mean ± SEM, and the analysis of variance was performed by ANOVA test and then by applying *post hoc* Bonferroni’s multiple comparison test. The *p*-values less than 0.05 were considered significant. Data were analyzed using the “Origin 9.1” software.

## Results

**FIGURE 1:**
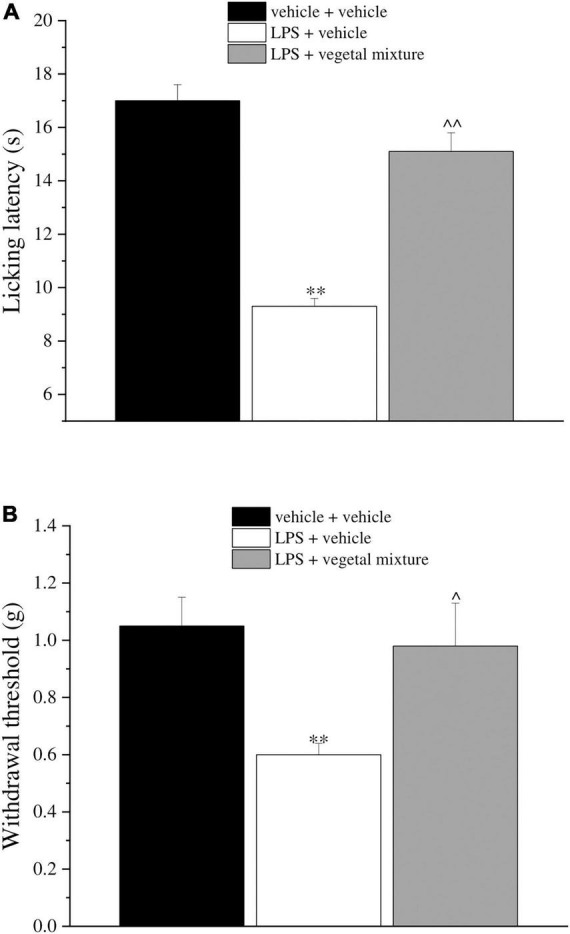
Effect of a vegetal mixture on LPS-induced thermal and mechanical allodynia. LPS (1 mg kg^– 1^) was intraperitoneally injected on days 1, 3, 5, and 8. The vegetal mixture was composed of *Zingiber officinale* (150 mg kg^– 1^), *Echinacea purpurea* (20 mg kg^– 1^), and *Centella asiatica* (200 mg kg^– 1^), suspended in 1% carboxymethylcellulose sodium salt (CMC) and orally administered daily starting from day 1 till the end of the experiment. Control animals were treated with vehicles. On day 9 and 24 h after the last administration of the vegetal mixture, **(A)** thermal and **(B)** mechanical allodynia was assessed by the Cold plate and von Frey tests, respectively. Data are expressed as the mean ± SEM of values from eight mice analyzed in two different experimental sets. ^**^p < 0.01 vs. vehicle + vehicle; ∧*p* < 0.05 and ∧∧*p* < 0.01 vs. LPS + vehicle.

**FIGURE 2:**
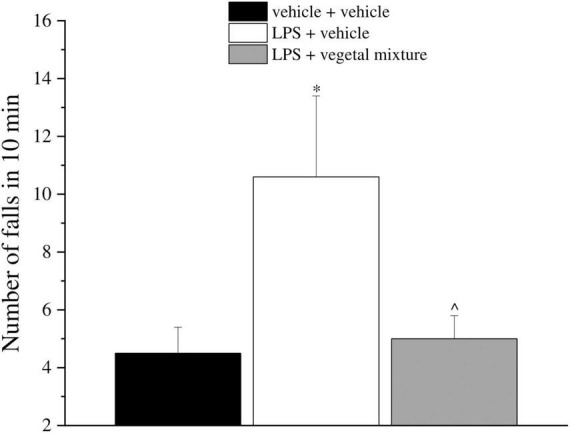
Effect of a vegetal mixture on LPS-induced motor alterations. LPS (1 mg kg^– 1^) was intraperitoneally injected on days 1, 3, 5, and 8. The vegetal mixture was composed of *Zingiber officinale* (150 mg kg^– 1^), *Echinacea purpurea* (20 mg kg^– 1^), and *Centella asiatica* (200 mg kg^– 1^), suspended in 1% carboxymethylcellulose sodium salt (CMC) and orally administered daily starting from day 1 till the end of the experiment. Control animals were treated with vehicles. Starting from day 9 and 24 h after the last administration of the vegetal mixture, the motor coordination was assessed by the Rota rod test. Data are expressed as the mean ± SEM of values from eight mice analyzed in two different experimental sets. *p < 0.05 vs. vehicle + vehicle; ∧*p* < 0.05 vs. LPS + vehicle.

**FIGURE 3:**
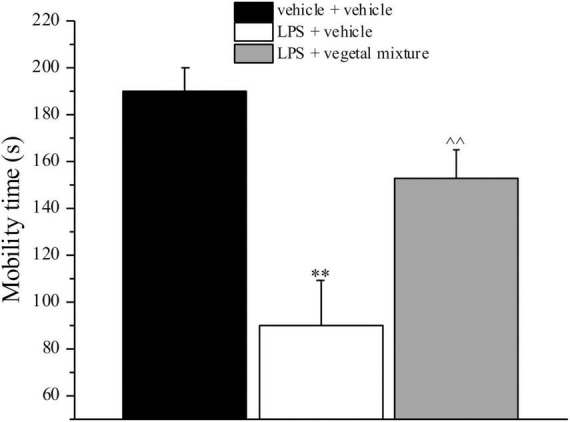
Effect of a vegetal mixture on LPS-induced depression-like behavior. LPS (1 mg kg^– 1^) was intraperitoneally injected on days 1, 3, 5, and 8. The vegetal mixture was composed of *Zingiber officinale* (150 mg kg^– 1^), *Echinacea purpurea* (20 mg kg^– 1^), and *Centella asiatica* (200 mg kg^– 1^), suspended in 1% carboxymethylcellulose sodium salt (CMC) and orally administered daily starting from day 1 till the end of the experiment. Control animals were treated with vehicles. Starting from day 9 and 24 h after the last administration of the vegetal mixture, the depression-like behavior was assessed by the Porsolt test. Data are expressed as the mean ± SEM of values from eight mice analyzed in two different experimental sets. ^**^p < 0.01 vs. vehicle + vehicle; ∧∧*p* < 0.01 vs. LPS + vehicle.

**FIGURE 4:**
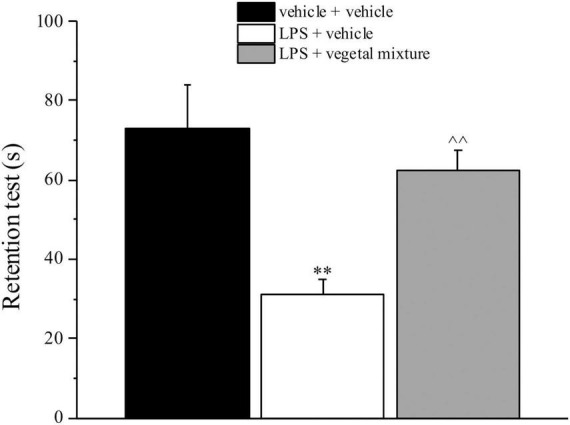
Effect of a vegetal mixture on LPS-induced memory loss. LPS (1 mg kg^– 1^) was intraperitoneally injected on days 1, 3, 5, and 8. The vegetal mixture was composed of *Zingiber officinale* (150 mg kg^– 1^), *Echinacea purpurea* (20 mg kg^– 1^), and *Centella asiatica* (200 mg kg^– 1^), suspended in 1% carboxymethylcellulose sodium salt (CMC) and orally administered daily starting from day 1 till the end of the experiment. Control animals were treated with vehicles. Starting from day 9 and 24 h after the last administration of the vegetal mixture, the memory was assessed by the Passive avoidance test. Data are expressed as the mean ± SEM of values from eight mice analyzed in two different experimental sets. ^**^p < 0.01 vs. vehicle + vehicle; ∧∧*p* < 0.01 vs. LPS + vehicle.

**FIGURE 5:**
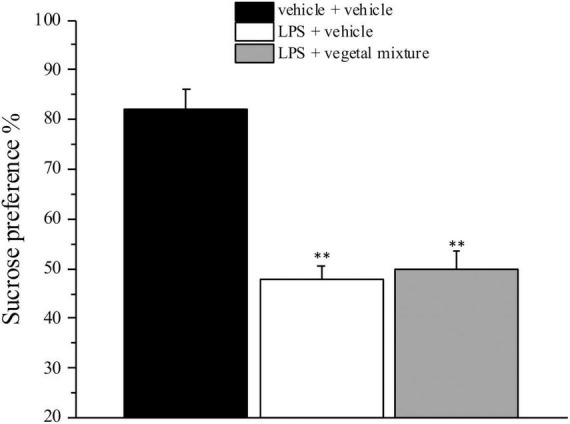
Effect of a vegetal mixture on LPS-induced anhedonia. LPS (1 mg kg^– 1^) was intraperitoneally injected on days 1, 3, 5, and 8. The vegetal mixture was composed of *Zingiber officinale* (150 mg kg^– 1^), *Echinacea purpurea* (20 mg kg^– 1^), and *Centella asiatica* (200 mg kg^– 1^), suspended in 1% carboxymethylcellulose sodium salt (CMC) and orally administered daily starting from day 1 till the end of the experiment. Control animals were treated with vehicles. Starting from day 9 and 24 h after the last administration of the vegetal mixture, anhedonia was assessed by the Sucrose preference test. Data are expressed as the mean ± SEM of values from eight mice analyzed in two different experimental sets. ^**^p < 0.01 vs. vehicle + vehicle.

**FIGURE 6:**
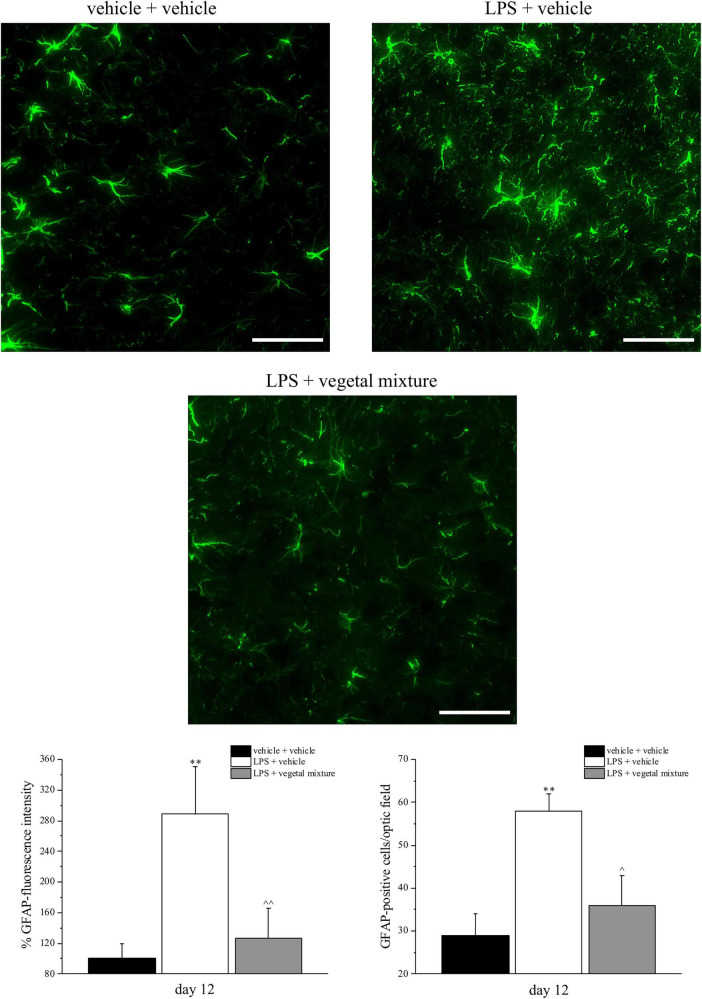
Effect of a vegetal mixture on LPS-induced astrocyte activation. LPS (1 mg kg^– 1^) was intraperitoneally injected on days 1, 3, 5, and 8. The vegetal mixture was composed of *Zingiber officinale* (150 mg kg^– 1^), *Echinacea purpurea* (20 mg kg^– 1^), and *Centella asiatica* (200 mg kg^– 1^), suspended in 1% carboxymethylcellulose sodium salt (CMC) and orally administered daily starting from day 1 till the end of the experiment. Control animals were treated with vehicles. At the end of the behavioral experiments and 24 h after the last administration of the vegetal mixture, animals were sacrificed and the lumbar spinal cord was collected. The number of GFAP-positive cells was measured in the dorsal horn of the L4–L5 spinal cord. Transverse sections of the spinal cord were imaged with a × 40 objective lens (scale bar = 50 μm). Histograms show the quantitative analysis of GFAP fluorescence intensity and the number of GFAP-positive cells/optic fields. Data are expressed as the mean ± SEM of values from eight mice analyzed in two different experimental sets. ^**^p < 0.01 vs. vehicle + vehicle; ∧*p* < 0.05 and ∧∧*p* < 0.01 vs. LPS + vehicle.

**FIGURE 7:**
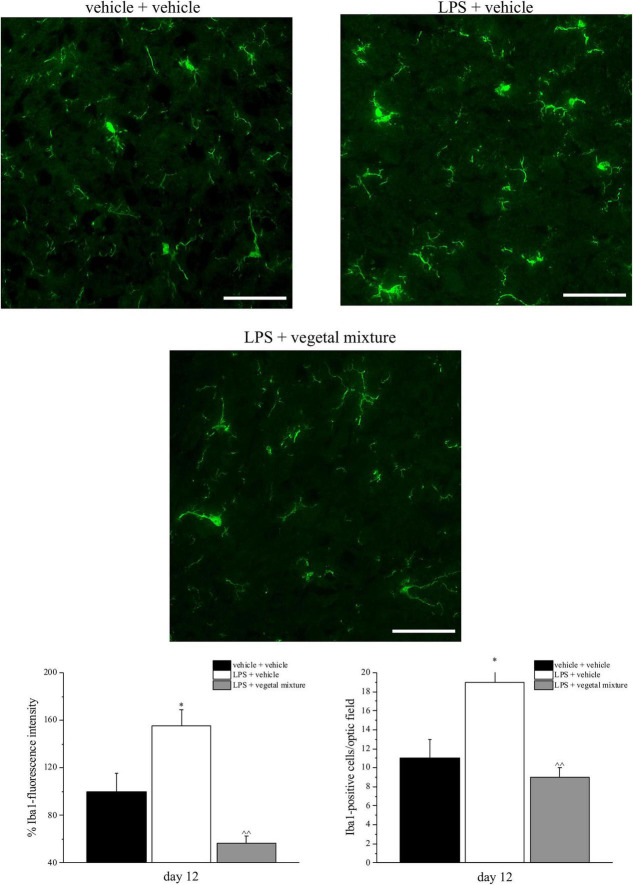
Effect of a vegetal mixture on LPS-induced microglial activation. LPS (1 mg kg^– 1^) was intraperitoneally injected on days 1, 3, 5, and 8. The vegetal mixture was composed of *Zingiber officinale* (150 mg kg^– 1^), *Echinacea purpurea* (20 mg kg^– 1^), and *Centella asiatica* (200 mg kg^– 1^), suspended in 1% carboxymethylcellulose sodium salt (CMC) and orally administered daily starting from day 1 till the end of the experiment. Control animals were treated with vehicles. At the end of the behavioral experiments and 24 h after the last administration of the vegetal mixture, animals were sacrificed and the lumbar spinal cord was collected. The number of Iba1-positive cells was measured in the dorsal horn of the L4–L5 spinal cord. Transverse sections of the spinal cord were imaged with a × 40 objective lens (scale bar = 50 μm). Histograms show the quantitative analysis of Iba1 fluorescence intensity and the number of Iba1-positive cells/optic fields. Data are expressed as the mean ± SEM of values from eight mice analyzed in two different experimental sets. *p < 0.05 vs. vehicle + vehicle; ∧∧*p* < 0.01 vs. LPS + vehicle.

**FIGURE 8:**
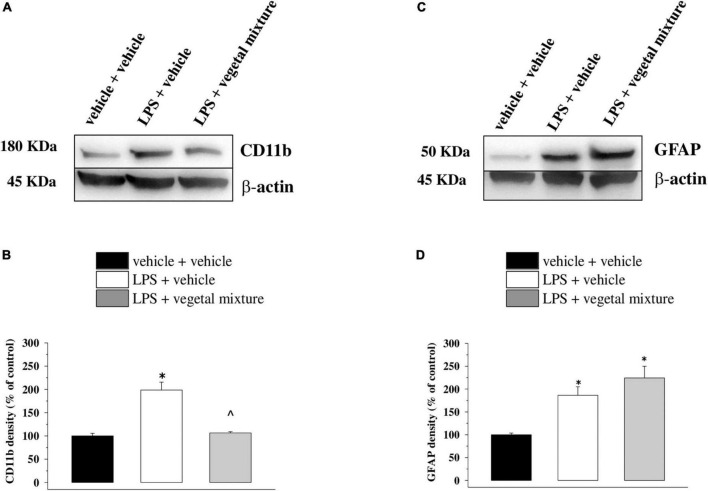
Effect of a vegetal mixture on LPS-induced microglia and astrocyte activation in the prefrontal cortex. LPS treatment and sacrifice of mice were carried out as described in the legend in Figure 7. The prefrontal cortex (PFC) was collected and analyzed for the density of the CD11b protein, a marker of microglia, and of GFAP, a marker of astrocytes. **(A)** Representative Western blot of the immunostainings for CD11b and β-actin, in vehicle-treated, LPS-treated, and LPS + vegetal mixture-treated mouse PFC lysates. The protein β-actin was used as an internal control. The blot is representative of the analysis of four animals for each experimental group. **(B)** Quantification of the change of CD11b density in the PFC in vehicle-treated, LPS + vehicle, and LPS + vegetal mixture-treated mice. Results are calculated as CD11b/β-actin ratio and are expressed as a percentage of the respective ratio in vehicle-treated mice. **(C)** Representative Western blot of the immunostainings for GFAP and β-actin, in vehicle-treated, LPS-treated, and LPS + vegetal mixture-treated mouse PFC lysates. The protein β-actin was used as an internal control. The blot is representative of the analysis of four animals for each experimental group. **(D)** Quantification of the change of GFAP density in the PFC of mice as described above, respectively. Data are expressed as mean ± SEM of eight mice analyzed in two different experimental sets. *p < 0.05 vs. vehicle-treated mice; ∧*p* < 0.05 vs. LPS-treated mice.

**FIGURE 9:**
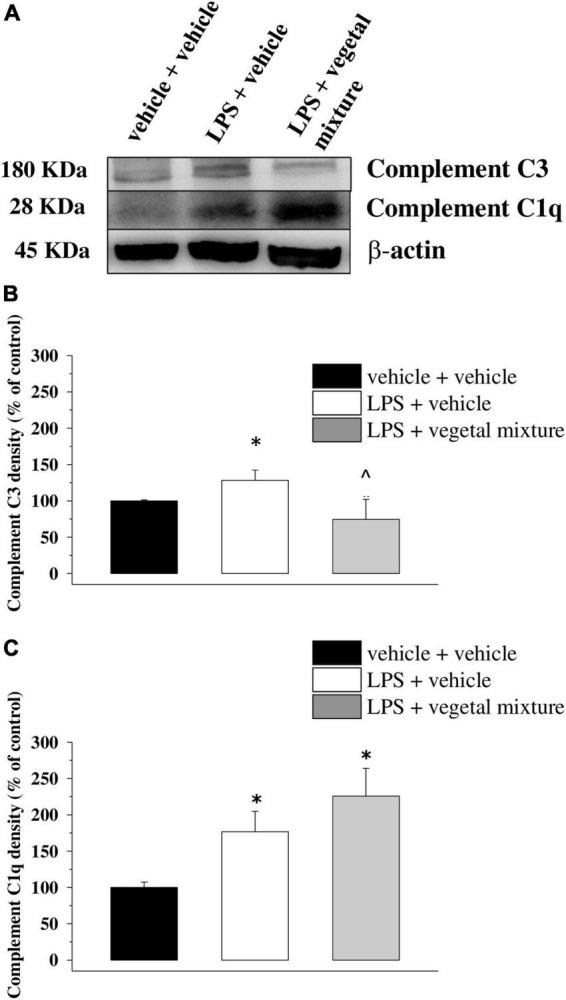
Effect of a vegetal mixture on LPS-induced overproduction of complement components in the prefrontal cortex. The prefrontal cortex (PFC) lysates were analyzed for the density of the C1q and the C3 proteins. **(A)** Representative Western blot of the immunostainings for C3, C1q, and β-actin, in the PFC lysates of vehicle-treated, LPS-treated, and LPS + vegetal mixture-treated mice. The protein β-actin was used as an internal control. The blot is representative of the analysis of four animals for each experimental group. **(B)** Quantification of the change of C3 density in the PFC of vehicle-treated, LPS + vehicle, and LPS + vegetal mixture-treated mice. Results are calculated as C3 ÷β-actin ratio and are expressed as a percentage of the respective ratio in vehicle-treated mice. **(C)** Quantification of the change of C1q density in the PFC of mice as above. Data are expressed as mean ± SEM of eight mice analyzed in two different experimental sets. *p < 0.05 vs. vehicle-treated mice; ∧*p* < 0.05 vs. LPS-treated mice.

**FIGURE 10:**
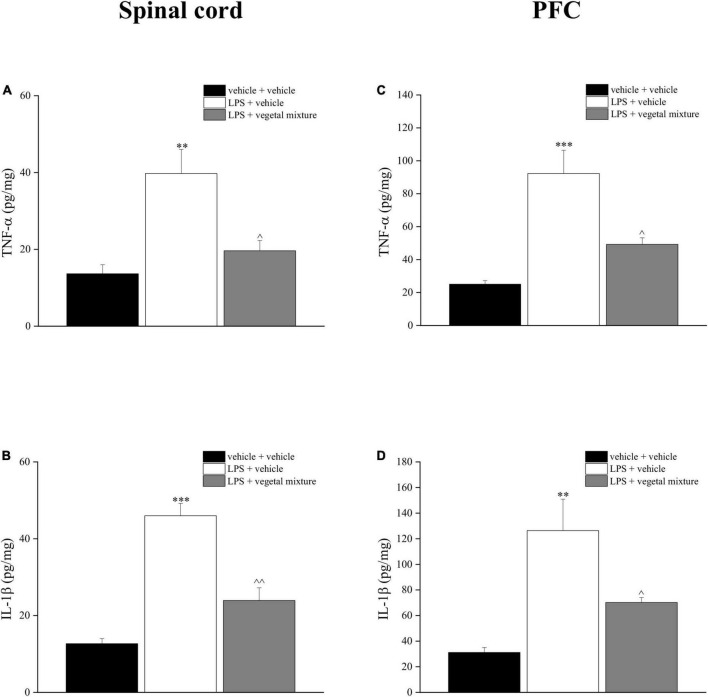
Effect of a vegetal mixture on LPS-induced overproduction of TNF-α and IL-1β levels in the prefrontal cortex and spinal cord. LPS (1 mg kg^– 1^) was intraperitoneally injected on days 1, 3, 5, and 8. The vegetal mixture was composed of *Zingiber officinale* (150 mg kg^– 1^), *Echinacea purpurea* (20 mg kg^– 1^), and *Centella asiatica* (200 mg kg^– 1^), suspended in 1% carboxymethylcellulose sodium salt (CMC) and orally administered daily starting from day 1 till the end of the experiment. Control animals were treated with vehicles. At the end of the behavioral experiments and 24 h after the last administration of the vegetal mixture, animals were sacrificed and the prefrontal cortex and the lumbar spinal cord were collected. **(A)** TNF-α and **(B)** IL-1β concentrations in the spinal cord of vehicle-treated, LPS-treated, and LPS + vegetal mixture-treated mice. **(C)** TNF-α and **(D)** IL-1β concentrations in the PFC of vehicle-treated, LPS-treated, and LPS + vegetal mixture-treated mice. Data are expressed as mean ± SEM of eight mice analyzed in two different experimental sets. ^**^p < 0.01 and ^***^p < 0.001 vs. vehicle-treated mice; ∧*p* < 0.05 and ∧∧*p* < 0.01 vs. LPS-treated mice.

Neuroinflammation was established by the sub-chronic injection of 1 mg kg^–1^ LPS (for a total of four administrations performed on days 1, 3, 5, and 8). The vegetal mixture was composed of dry extracts obtained from *Zingiber officinale* (150 mg kg^–1^), *Echinacea purpurea* (20 mg kg^–1^), and *Centella asiatica* (200 mg kg^–1^) and was daily *per os* administered from day 1 till the end of the behavioral experiments. From day 9 onward, several symptoms of neuroinflammation were analyzed. As reported in Figure 1, LPS induced a significant decrease in the animal’s pain threshold measured by thermal and mechanical non-noxious stimuli (Cold plate and von Frey tests, respectively). Indeed, on day 9, the licking latency significantly decreased from a value of about 17 s in the control group to about 9 s in the animals treated with LPS when the mice were exposed to a cold surface at 4°C (Cold plate test; Figure 1A). The daily administration of the vegetal mixture was able to counteract the thermal allodynia caused by LPS. One point to be noted is that the results were obtained 24 h after the last treatment with the mixture and were not evoked by the daily acute administration. Similarly, the paw stimulation with a non-noxious mechanical stimulus allowed us to observe a decrease in the withdrawal response in LPS-treated mice that was recovered in the group treated with LPS + vegetal mixture (Figure 1B). The LPS treatment also induced motor incoordination which was evaluated by the Rota rod test. The results showed a massive increase in the number of falls from the rod during 10 min in comparison to the vehicle + vehicle-treated animals. The combination of *Zingiber officinale, Echinacea purpurea*, and *Centella asiatica* improved the mouse performance determining a significant reduction in the number of falls, which was evident by the scores similar to those measured in the control animals (Figure 2). With regard to the impairments affecting memory and mood, we analyzed the depression-like behavior by the Porsolt test, anhedonia by the sucrose preference test, and memory by the passive avoidance test. In the Porsolt test, repeated administration of LPS caused a reduction in the mobility time after the mice were placed in a cylinder filled with water from where they could not escape. The treatment with the vegetal mixture significantly increased this parameter, reaching a value of about 150 s in comparison to 90 s observed in the LPS-treated animals (Figure 3). Moreover, the extracts were shown to reduce the amnesic effects of LPS in the passive avoidance test (Figure 4). The repeated administration of the vegetal mixture significantly antagonized the cognitive impairments induced by the lipopolysaccharide. In the retention test, the time spent in the light box was 73 ± 11 s for vehicle + vehicle group, 31 ± 4 s for LPS + vehicle group, and 103 ± 6 s for LPS + vegetal mixture-treated animals (Figure 4). Anhedonia, the incapacity to experience pleasure, is a hallmark symptom of several nervous system diseases. Anhedonia was induced by LPS injections and was evaluated as a significant reduction in sucrose intake in comparison to control animals; however, the treatment with the vegetal mixture was unable to restore this parameter (Figure 5). At the end of the behavioral measurements, the dorsal horn of the lumbar spinal cord was analyzed. Four LPS injections induced a significant increase in the number of GFAP and Iba1-positive cells (Figures 6, 7, respectively). Also, the fluorescence intensity of astrocytes and microglia was upregulated by LPS in comparison to the control group. The vegetal mixture prevented the activation of glial cells, as shown in Figures 6, 7. LPS treatment also caused a significant increase in the density of CD11b, a specific marker of microglia and macrophages, and GFAP in the PFC of the inflamed animals, which suggests that microgliosis and astrocytosis also occur in this region (Figure 8). The vegetal mixture recovered the CD11b signal to the level detected in the vehicle-treated animals (Figures 8 A,B), but did not affect the GFAP density, which was unmodified with respect to LPS-treated mice (Figures 8C,D). In recent years, the involvement of the complement system in the progression of neurodegenerative and neuroinflammatory disorders emerged in the interest of researchers. Because of the huge increase in the CD11b, which selectively binds the C3 component of the complement system, we determined whether LPS could also impact the expression of the C1q and the C3 complement proteins, i.e., those complement components that are mainly involved in the classic and the alternative pathways of the activation of the immune complex complement cascade and that are associated to the mechanisms of synaptic pruning and remodeling of central synapses (44, 45). Both C1q and C3 proteins were over-expressed in the PFC of the LPS-treated mice. The vegetal mixture significantly reduced the C3 overproduction, leaving unaltered the density of the C1q protein (Figure 9). Finally, the mixture was also able to significantly reduce the overproduction of TNF-α and IL-1β cytokines in the PFC and spinal cord, which were found to be increased in the LPS-treated animals (Figure 10).

## Discussion

Neuroinflammation has been implicated as a pathological contributor in several diseases affecting the nervous system, and alleviation of neuroinflammation is believed to reduce disease severity and improve patient outcomes in the majority of cases. Diseases involving the nervous system and different neurodegenerative syndromes, excluding those simply associated with aging, are currently one of the great problems in global health, and there is no doubt that neuroinflammation is involved to a lesser or greater extent in most brain pathologies, whether chronic, neurodegenerative, or acute brain damage, for a multitude of different causes. To note, recent studies have highlighted that SARS-CoV-2 infection also induces neuroinflammation with severe long-term consequences (46) due to the evoking of cytokine storm (47).

In this work, we highlighted the efficacy of a vegetal mixture composed of *Echinacea Purpurea*, *Centella asiatica*, and *Zingiber officinale* in a mouse model of neuroinflammation evoked by the sub-chronic injection of LPS. In particular, the mixture was able to counteract pain, depression, and motor and memory impairments, all pathologies which are associated with neuroinflammation. Moreover, *ex vivo* analysis demonstrated the efficacy of the treatment in reducing glial cell activation in the spinal cord and in stabilizing the central component proteins that were increased by LPS.

It has been described that systemic infection is associated with an increased frequency of behavioral and cognitive complications (48, 49). The stimulation of the peripheral immune system in mice evoked massive neuroinflammation, which results in prolonged sickness (10), impaired working memory (50), and depressive-like behavior (51).

In this context, it is crucial to design new therapies that can simultaneously act on various aspects, such as improving the recovery from symptoms, controlling the inflammatory and antioxidant processes, and modulating the immune response to attenuate the progression of the disease.

Over the years, the phytotherapeutic approach has been shown to be a valid strategy against human diseases and disorders. The plants were used for medicinal purposes since ancient times because the transcripts describe their effectiveness against various disorders. Several epidemiological studies reported a close correlation between the intake of a diet rich in antioxidant phytochemicals and a lower incidence of age-related neurological disorders for which inflammation is the core pathophysiological mechanism (52). In this study, the neuroinflammatory processes were evoked by the sub-chronic injection of LPS, a toll-like receptor 4 (TLR-4) ligand (53). TLR-4 is primarily expressed in microglia in the CNS (54), and its activation determined the production of proinflammatory cytokines, prostaglandins, and nitric oxide (NO) (55, 56), the key mediators of the neuroinflammatory processes. After four injections of LPS, mice reported mechanical and thermal allodynia as measured by the von Frey and Cold plate tests, confirming that inflammation causes an alteration in the mouse pain threshold and induces pathological hypersensitivity, which is the first step from physiological nociception to persistent pain (57). Concomitantly, animals developed behavioral symptoms predictive of mood and cognitive alterations that were largely prevented by the vegetal treatment. All the pain-relieving effects evoked by the treatment can be attributed to the complexity of the bioactive compounds present in the vegetal mixture. An important phenolic component emerged from the phytochemical characterization, in particular, *Zingiber officinale* extract contained gingerols (3.107%) and 6-shogaol (0.647%), which have been found to be effective in alleviating inflammation, especially in inflammatory bowel diseases (58, 59), and in reducing pain evoked by formalin and acetic acid injection in mice (60). In this context, LPS caused an overproduction of proinflammatory cytokines, such as TNF-α and IL-1β in the prefrontal cortex and spinal cord regions, which was counteracted by the vegetal mixture. A study by Ha and colleagues demonstrated the neuroprotective role of 6-shogaol in a BV-2 and primary microglial cell cultures stimulated by LPS and in an *in vivo* model of neuroinflammation. In particular, these effects seem to be related to both anti-inflammatory and antioxidant properties exerted by 6-shogaol on activated microglia, the resident immune cells of the nervous system (61). *Centella asiatica* is characterized by the presence of 2.6% phenols, mainly constituted by flavonoids and phenylpropanoid derivatives (34). This extract has already been demonstrated to possess anti-hyperalgesic properties in a rat model of osteoarthritis and reduce the NO imbalance in a cell line of macrophages stimulated by LPS, thus highlighting the antioxidant capacity of these compounds (34). In addition, Centella is enriched in polyphenols, including rosmarinic acid which possesses anti-inflammatory activities (62, 63) because of its ability to positively control the complement cascade pathways (64). Moreover, extensive literature reported the neuroprotective role of Centella asiatica. Indeed, its oral supplementation resulted in a strong memory enhancement effect in the preclinical studies (65), and the positive modulation of cognitive functions has also been confirmed in elderly volunteers (66). In an aged Alzheimer’s disease mouse model, water extract of *Centella asiatica* improved cognitive functions *via* increasing the Nrf2 gene expression in the hippocampus and by reducing Aβ plaque-associated SOD1 in the hippocampus and cortex (67).

*Echinacea purpurea* extract is predominantly (6%) constituted by phenolic compounds (0.037% chlorogenic acid, 3.825% cicoric acid, and 2.083% caftaric acid), which exhibit immunomodulatory and antioxidant properties (28). It is important to mention that Echinacea preparations, particularly obtained from *E. purpurea*, have been proposed to be used as a preventive treatment or therapeutic adjuvant against COVID-19 and as an immune-boosting agent in people at risk or with comorbidities, owing to its plausible effects on immunity, infection, and inflammation (68). Reports regarding the enhancement of immune function suggest that this effect is related to increased secretion of several cytokines by monocytes, including TNF-α, IL-1, IL-6, and IL-10 (69). Even if each extract produces effective results on its own, the mixture contains dozens of compounds with different therapeutic effects that act by different mechanisms, thus making possible a manifold approach to the complexity of the disease evoked by LPS.

The alteration in the mouse pain threshold was not the only symptom caused by LPS, but it also caused mood disorders like depression and anhedonia, and memory and motor impairments. All these alterations were counteracted by the treatment with plant extract except for anhedonia. A number of studies indicate how neuroinflammation is linked to the neurotransmitter acetylcholine which helps organisms to filter the enormous amounts of information received from the environment.

Particularly, in neuroinflammation, the α7 nicotinic acetylcholine receptor (α7 nAChR) is mostly involved, since it regulates cognitive functions and inflammatory reactions.

In the sensory cortex, it acts by fine-tuning the activity of neurons to enhance attention, which subsequently helps with learning and memory (70–72). Patients with neurodegenerative diseases exhibit a lower expression of acetylcholinesterase neurons, choline acetyltransferase, and acetylcholine synthesis, release, and re-uptake. Moreover, it is well-known how α7 nicotinic acetylcholine receptor (nAChRs) plays an important role in the management of neuropathic pain (73–75). It has been demonstrated that systematic injections of LPS reduced the density of α7 nAChRs in the brain that physiologically promote the internalization of processed Aβ forms (76, 77). Subsequently, the decrease in the density of α7 nAChR on the plasma membrane impairs Aβ metabolism and promotes the accumulation of extracellular Aβ (78, 79); this might be the mechanism by which LPS leads to neuroinflammation and cognitive impairments. It has been reported by the literature that zingiber extract restores acetylcholine levels and exhibits protective ability and cognitive enhancing properties during ethanol withdrawal (80), as well as *Centella asiatica* extract inhibits acetylcholinesterase, inflammation, and oxidative stress activities (81). These findings support the data obtained in this study regarding the efficacy of the vegetal mixture on all symptoms analyzed.

The important cell population in the CNS is represented by glial cells, predominantly microglia and astrocytes (82). Among their functions are the regulation of PH and ionic balance for the maintaining of homeostasis, uptake and degradation of neurotransmitters, and modulation of neuroinflammation in physiological and pathological conditions (83, 84). Several studies pointed out the pivotal role of glial cells in the generation and preservation of chronic pain (85, 86). Also, neuropathic (87) and pharmacological strategies to suppress the activity of microglial cells and astrocytes are being explored as therapies demonstrating a valid option in the management of chronic and neuropathic pain conditions (87). Although LPS may induce the activation of satellite glial cells in the dorsal root ganglia (88), the importance of glial cell activation in the spinal cord has recently been suggested (89, 90). In LPS-treated mice, we recorded an increase in the number of GFAP-positive cells and upregulation of GFAP expression, as well as an increase in the number of Iba1-positive cells and upregulation of Iba1 expression. The maladaptive plasticity of the glial cells in the spinal cord was attenuated by the treatment with the vegetal mixture, thus highlighting the importance of the inhibition of these cell populations in the control of neuroinflammation. Astrocytosis and microgliosis also emerged in the PFC and could be predictive of amnesic symptoms observed in LPS-treated mice. Activated microglia and astrocytes are also central sources of the complement proteins, particularly C1q and C3 proteins, which in physiological conditions regulate neuronal development by suppressing weak synapses, but if aberrantly expressed, exacerbate synaptic derangements and central inflammation, contributing to the cognitive and mood decline associated with aging or to neurodegenerative disorders, i.e., Alzheimer’s disease (91, 92).

In this context, a significant reduction of the C3 density in the PFC of the LPS-treated mice receiving the mixture could be predictive of anti-inflammatory activity and the amelioration of the cognitive performances observed in *in vivo* studies. The positive impact of the vegetal mixture is of even more interest considering the concomitant persistence of a high level of C1q, which if over-expressed in the absence of other complement components modulates the gene expression critical for neuronal survival (93).

## Conclusion

In conclusion, the present work demonstrated the efficacy of daily treatment with a vegetal mixture composed of *Zingiber officinale*, *Centella asiatica*, and *Echinacea purpurea* extracts in the control of neuroinflammation induced by LPS in mice. In particular, mood disorder, pain, and motor and cognition impairments were counteracted. Moreover, the vegetal mixture induced a reduction in the activation of glial cells in the spinal cord and PFC. The results obtained in this study represent an important milestone for the use of phytotherapies in the control of neuroinflammation.

## Data availability statement

The raw data supporting the conclusions of this article will be made available by the authors, without undue reservation.

## Ethics statement

Formal approval to conduct the experiments described was obtained from the Animal Subjects Review Board of the University of Florence.

## Author contributions

LM, CG, and LD: conceptualization. CP, JL, EL, and AT: methodology. CC and VF: software. LM and EL: validation. GO: formal analysis. MB and DC: resources. LM and AP: data curation. LuM: original draft preparation. LuM, CG, LD, SP, and AP: reviewing and editing. CG, AP, and LD: supervision. CG and LD: project administration. CG: funding acquisition. All authors have read and agreed to the published version of the manuscript and have contributed substantially to the work reported.

## Footnotes

1
https://imagej.nih.gov/
